# Central Precocious Puberty in a Child With Metachromatic Leukodystrophy

**DOI:** 10.3389/fendo.2018.00497

**Published:** 2018-08-24

**Authors:** Gilda Belli, Emanuele Bartolini, Andrea Bianchi, Mario Mascalchi, Stefano Stagi

**Affiliations:** ^1^Department of Health Sciences, University of Florence, Anna Meyer Children's University Hospital, Florence, Italy; ^2^Neurology Unit and Laboratories, Anna Meyer Children's University Hospital, Florence, Italy; ^3^Neurology Unit, San Luca Hospital USL Nord-ovest Toscana, Lucca, Italy; ^4^Pediatric Neuroradiology, Anna Meyer Children's University Hospital, Florence, Italy; ^5^Department of Experimental and Clinical Biomedical Sciences “Mario Serio”, University of Florence, Florence, Italy

**Keywords:** metachromatic leucodystrophy, central precocious puberty, precocious puberty, metabolic disease, white matter disease

## Abstract

Metachromatic leucodystrophy (MLD) is a rare inherited lysosomal disorder caused by reduced activity of the enzyme arylsulfatase A with accumulation of sulfatides in the nervous system. We report a female child affected by MLD who developed central precocious puberty (CPP). This association has not been described so far. The proposita, after normal growth and psychomotor development, at age of 30 months presented with a rapidly progressive gait disturbance with frequent falls and with loss of acquired language skills. Magnetic resonance imaging showed leukoencephalopathy. Biochemical blood essays showed a 91% reduction in the arylsulfatase A activity and genetic analysis revealed compound heterozygous mutations of the *Arylsulfatase A* gene, enabling diagnosis of MLD. Subsequently, the patient had further rapid deterioration of motor and cognitive functions and developed drug-resistant epilepsy. At 4 years and 7 months of age bilateral thelarche occurred. Magnetic resonance imaging showed a small pituitary gland, extensive signal changes of the brain white matter, increased choline, decreased N-acetyl-aspartate and presence of lactate on ^1^HMR spectroscopy. Pelvic ultrasound demonstrated a slightly augmented uterine longitudinal diameter (42 mm). The gonadotropin-releasing hormone stimulation test revealed a pubertal LH peak of 12.9 UI/l. A diagnosis of CPP was made and treatment with gonadotropin-releasing hormone agonists was initiated, with good response. In conclusion, a CPP may occur in MLD as in other metabolic diseases with white matter involvement. We hypothesize that brain accumulation of sulfatides could have interfered with the complex network regulating with the hypothalamic-pituitary axis and thus triggering CPP in our patient.

## Introduction

Metachromatic leucodystrophy (MLD; OMIM #250100) is a rare autosomal recessive inherited lysosomal disorder, caused by reduced activity of the enzyme arylsulfatase A, due to mutations in the *Arylsulfatase A* gene (*ARSA*; OMIM ^*^ 607574) that is located on chromosome 22q13.33 (1, 2). Rarely, the phenotype is also due to mutations in the *Prosaposin* gene (*PSAP*; OMIM ^*^ 176801), encoding an activator of ASA (prosaposin), or in *sulfatase-modifying factor-1* gene (*SUMF1*; OMIM ^*^ 607939) (2, 3). Birth prevalence of MLD is estimated between 1.4 e 1.8 per 100.000 (4).

Arylsulfatase A deficiency results in accumulation of sulfatides in the central and peripheral nervous system, typically leading to demyelination of the brain white matter (1). Psychomotor regression, intellectual and behavioral changes are the main clinical features of MLD (4, 5).

According to the age of onset, the disease is classified in three clinical subtypes: (a) late-infantile form (before 30 months), characterized by rapidly progressive psychomotor regression, ataxia and areflexia; (b) juvenile variant (onset between 2.5 and 16 years of age), often beginning with impaired fine motor skills, deterioration of school performance or behavior abnormalities; c) adult onset type (after the age of 16 years), with memory deficits or emotional instability as the most frequent initial symptoms (1, 5).

Herein we report the case of a girl affected by MLD who developed central precocious puberty (CPP). This association has not been described so far. Written informed consent was obtained from the parents of the patient for the publication of this case report.

## Case presentation

This female child was diagnosed with juvenile variant of MLD at 2.5 years of age. She had exhibited normal growth and psychomotor development. At age of 30 months she presented with progressive gait disturbance with frequent falls as well as with loss of acquired language skills, which worsened despite psychomotor rehabilitation. Brain Magnetic resonance imaging (MRI) revealed a marked and widespread hyperintensity of white matter in T2 weighted images, consistent with a leukodystrophy. Biochemical blood essays showed a 91% reduction in the arylsulfatase A activity, consistent with the diagnosis of MLD, which thereafter was confirmed by genetic analysis. Subsequently, a rapid deterioration of motor and cognitive functions occurred, that was associated with drug-resistant epilepsy on polytherapy with valproate, lamotrigine, and levetiracetam.

At 4 years and 7 months of age, bilateral thelarche was detected at physical examination and the neurological assessment showed spastic tetraparesis and severe intellectual disability. Family history of precocious puberty was negative. At endocrinological evaluation, human chorionic gonadotropin (<1.00 mIU/ml), alpha-fetoprotein (3.10 IU/ml) and carcinoembryonic antigen (1.90 μg/L) resulted in the normal range. The hormonal profile showed dehydroepiandrosterone sulfate in normal range according to age (<0.4071 μmol/L), but the patient showed moderately augmented levels of 17-Hydroxyprogesterone (5.80 nmol/L) and 17 beta-estradiol (215.51 pmol/L). Thyroid function was normal (TSH 2.64 mIU/L, normal range 0.40–4.00 mIU/L; FT4 12.74 pmol/L, normal range 10.30- 24.46 pmol/L; fT3 6.07 pmol/L, normal range 2.46–7.37 pmol/L). Also plasmatic levels of valproic acid were within the normal range (517.09 μmol/L; therapeutic range 346.71–693.43 mg/L). 25-hydroxyvitamin D was deficient (39.93 nmol/L; normal value > 74.88 nmol/L), while 1,25-dihydroxyvitamin D_3_ was within the normal range (98.40 pmol/L; normal range: 47.76–160.81 pmol/L). Ultrasonography excluded ovarian and adrenal masses and showed ovaries of prepubertal volume and uterus with infantile features. X-ray of the left wrist and hand revealed a bone age of 5 years, according to Greulich and Pyle method (6). At that time, we decided to postpone the gonadotropin-releasing hormone (GnRH) stimulation test, given the underlying disease and the absence of overt signs of pubertal activation.

**Figure 1:**
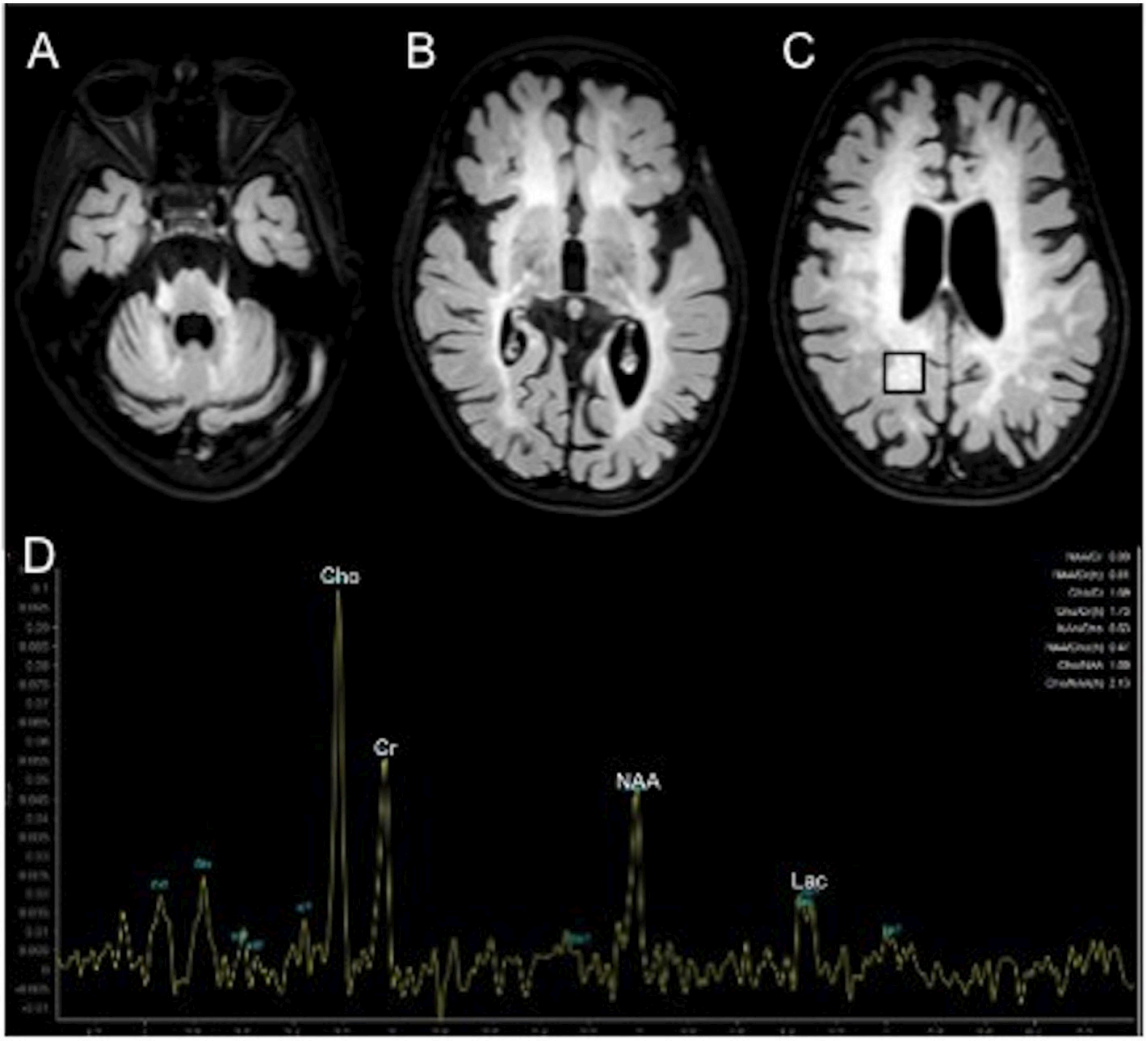
Contrast enhanced brain MRI **(A–D)**. Axial T2 weighted FLAIR images show extensive areas of increased signal symmetrically involving the middle cerebellar peduncles and cerebellar peridentate white matter **(A)** and the cerebral white matter **(B,C)**.^1^H MR spectroscopy in the affected right parietal white matter (black box in **C**) shows increased peaks of choline (Cho) and lactate (Lac) and decreased peak of N-acetyl-aspartate (NAA).

However, at the endocrine follow-up, when the patient was 4.8 year-old, she exhibited a progression of the pubertal staging (Ph3 B2-3, no axillary hair), according to Marshall and Tanner criteria (7). Ultrasonography showed a slight increase of the uterine longitudinal diameter (42 mm, normal values <40 mm) (8). She weighted 15.6 Kg [−1.31 SDS, 10th percentile according to Cacciari et al. (9)] and was 107 cm tall [−0.37 SDS, 25–50th percentile according to Cacciari et al. (9)]. Due to the suspicion of CPP, we performed a GnRH stimulation test with intravenous administration of 100 μg/m^2^ (maximum 100 μg) synthetic GnRH (Lutrelef 0.8 mg/10 mL, FerringS,p.A., Italy) (10) that revealed a pubertal LH peak of 12.90 IU/l. A detectable estradiol level (129.9655 pmol/L) confirmed the activation of gonadotropic axis (11). However, an ACTH stimulation test, carried out because of the mildly elevated value of 17-Hydroxyprogesterone, was normal. Values of cortisol (587.58 nmol/L at 08 AM, normal range 137.93–689.65 nmol/L) and androstenedione (3.40 nmol/L, normal range 1.00–11.50 nmol/L) were normal. Testosterone was undetectable (<0.6934 nmol/L). A contrast enhanced brain MRI (Figure 1) showed a small hypophysis without focal abnormalities, extensive symmetric areas of increased T2 signal in the cerebral white matter including the internal capsule, the cerebral peduncles, middle cerebellar peduncles and the cerebellar peridentate white matter and marked brain atrophy, consistent with MLD at an advanced stage. No contrast enhancement of the affected white matter was observed. Proton MR spectroscopy in the affected right parietal white matter showed increase of the choline, a decrease of the N-acetylasparate, and a peak of lactate (Figure 1). A diagnosis of CPP was made and she started treatment with GnRH agonists (triptorelin intramuscularly at a dosage of 1.875 mg every 28 days). She experienced a good response in the absence of significant adverse effects. Accordingly, after 6 months of therapy, a GnRH stimulation test showed a LH peak concentration of 1.42 IU/l and a reduced value of estradiol (80.03 pmol/L).

### Laboratory methods

All laboratory endocrinological measurements were determined by means of chemiluminescent immunometric assays with the use of commercially available kits for the Immulite 2000 systems analyzer (Siemens Healthcare Diagnostics). However, 17-Hydroxyprogesterone and androstenedione were assayed by radioimmunoassay (Diagnostic systems Laboratories and DIAsource, Nivelles, Belgium).

## Discussion

In MLD the accumulation of sulfatides into lysosomes of the oligodendrocytes, Schwann cells, phagocytes, astrocytes and neurons leads to widespread demyelination and inflammatory changes causing apoptosis in the central and peripheral nervous system (1, 12). MRI in MLD shows symmetric high T2 signal intensity in the cerebral white matter, sparing subcortical U fibers and in the corpus callosum. In more advanced stages, cerebellar white matter, thalami and basal ganglia can be involved. Typical, but not specific, is the so called “tigroid pattern” with radiating stripes of normal signal intensity within the T2 hyperintense white matter (1, 12).

Our case is peculiar because of the occurrence of CPP in a patient suffering from a severe and rapidly progressive form of MLD. To our knowledge, this is the first reported case of CPP in MLD. CPP is known to occur in patients with a variety of central nervous system (CNS) disorders (11, 13, 14), underlining the importance of early diagnostic work-up in these patients.

Notably, review of the literature revealed a handful of cases of inherited metabolic diseases implying damage of the brain white matter associated with CPP or other disorders of pubertal development (Supplementary Table 1).

For example, in mucopolysaccaridosis, a class of multi-organ dysfunction disorders due to progressive glycosaminoglycans accumulation, five cases of CPP were reported in patients affected by mucopolysaccharidosis type IIIA (SanFilippo disease) (15, 16). In Hurler syndrome (mucopolysaccharidosis type IH or MPS IH), due to α-L-iduronidase activity deficiency, CPP has been exceptionally described in patients after hematopoietic stem cell transplantation (17), but it is difficult to ascertain if it was an effect of hematopoietic stem cell transplantation or was innate to mucopolysaccharidosis type IH.

In Tay-Sachs disease, precocious puberty has been described, probably due to hypothalamic involvement (18). A case of CPP in a femal child of 4 years has been reported in more detail (19). In this case, autopsy revealed storage of GM2 gangliosides in the hypothalamus but not in pituitary gland, thus PP was attributed either to interruption of inhibitory influences on the pituitary-gonadal axis or to lesions that disrupt nerve endings storing LH-RH in the basal hypothalamus, resulting in a direct stimuli for LH-RH release. The author also suggests that the prolonged survival of patients with storage disorders may be one the main reasons why some of these patients have recently been described with precocious puberty (19).

In Phenylketonuria, two cases of CPP were reported so far. In the former, the author suggested a toxic metabolic effect of high serum phenylalanine levels as the trigger of premature activation of the hypothalamic-pituitary axis (20); in the latter, values of phenylalanine were persistently within the recommended range, therefore the rare association was considered to be coincidental (21).

In these reports, overall, a damage due to accumulation of toxic metabolites in brain or in blood is frequently hypothesized as the pathogenic mechanism leading to precocious puberty or other premature signs of puberty, although the existence of a causal relationship often could not be ascertained due to the paucity of data about pubertal development in these disorders.

Admittedly, because of its rarity, the association of CPP and MLD we observed could be coincidental. On the other hand, the rarity of incidence of CPP in MLD could reflect the low survival due to the severity of CNS involvement.

The onset of hypothalamic-hypophyseal-gonadal axis function is dependent upon the highly controlled and dynamic interactions among regulatory signals from the brain, pituitary and gonads (22). In addition, the puberty onset also requires a reciprocal communication neuron-glia which involves the action of two main excitatory amino acids: glutamate and aspartate. Besides that, some growth factors and the coordinated action of these neuroexcitatory amino acids, and specific genes, all of which representing a higher level of control governing the pubertal process, are also required in order for puberty to occur (22). So, as suggested in other diseases, we can hypothesize that sulfatides brain accumulation in our patient may have acted on the complex network that regulates GnRH secretion in the diencephalon that involves kisspeptin, neurokinin B, makorin ring finger protein 3, and others (23, 24), resulting in an enhancement of activators and suppression of inhibitors of gonadotropin releasing factors secretion, thus triggering puberty initiation (23, 24). Interestingly, some authors have reported a significant reduction of N-acetylasparate in the left dorsolateral prefrontal cortex of a group of bipolar patients with early puberty compared to controls (25). This aspect was reported also in our previous research about a young girl with NF1 who developed CCP related to a hamartoma of the tuber cinereum at 5 years of age (26). In our case, proton MR spectroscopy showed decrease of the N-acetylasparate, thus suggesting its possible pathogenic role in the initiation of puberty in our patient.

However, other hypothesis may be not excluded, as well as a concomitant role of the antiepileptic treatment in the pathogenesis of CPP. Although the endocrine effects of antiepileptic drugs have widely been investigated (27), only one case of precocious puberty has been reported in a child with myoclonic seizures treated with valproate (28). The authors hypothesized a causal relationship based on the stabilization of pubertal progression at withdrawal of valproate without any hormonal treatment. However they also suggested that epilepsy and CPP could be primitively associated. Indeed, a relationship between idiopathic CPP and not specific organic and functional alterations of CNS, disrupting the hypothalamic-pituitary-gonadal axis, has been postulated (28, 29).

## Concluding remarks

In conclusion, we report the first case of CPP occurrence in a patient affected by MLD. Although several CNS diseases including inherited metabolic diseases causing damage to the brain white matter can be associated with CPP or delayed puberty, the pathogenic link between these conditions and CPP is obscure. Nevertheless, our case underlines the possible occurrence of CPP, even in the first years of life, in patients suffering from neuro metabolic disorders; therefore, it suggests that pediatricians should be aware of this possibility and evaluate pubertal stage on a routine basis during follow up visits, referring patients to a pediatric endocrinologist and endocrine work up if any signs of pubertal development occurred. Larger multicenter studies are needed, enrolling patients suffering from MLD with different disease severity, despite the early death of some of these, to assess the time of onset of puberty in this population and to better clarify the relationship between MLD and CPP.

## Author contributions

GB conceptualized and designed the work and drafted the initial manuscript. SS, MM, AB, and EB conceptualized, designed the work and reviewed and revised the manuscript. All authors approved the final manuscript as submitted and agree to be accountable for all aspects of the work.

### Conflict of interest statement

The authors declare that the research was conducted in the absence of any commercial or financial relationships that could be construed as a potential conflict of interest.

## Abbreviations

MLD: Metachromatic leucodystrophy
CPP: Central precocious puberty
MRI: Magnetic resonance imaging
GnRH: Gonadotropin-releasing hormone
CNS: Central nervous system.
