# Supplementary material for: Central Precocious Puberty in a Child With Metachromatic Leukodystrophy
Source: Front Endocrinol (Lausanne). 2018 Aug 24;9:497. doi: 10.3389/fendo.2018.00497 (PMC6117375; doi:10.3389/fendo.2018.00497)
Supplement: Supplementary file 1 [file Table_1.DOCX]

Supplementary Material

Central precocious puberty in a child with metachromatic leukodistrophy

**Gilda Belli^1*^, Emanuele Bartolini^2,3^, Andrea Bianchi^4^, Mario Mascalchi^4,5^, Stefano Stagi^1^**

^1^Department of Health Sciences, University of Florence, Anna Meyer Children’s University Hospital, Florence, Italy;

^2^Neurology Unit and Laboratories, Anna Meyer Children’s University Hospital, Florence, Italy;

^3^Neurology Unit, San Luca Hospital USL Nord-ovest Toscana, Lucca, Italy;

^4^Pediatric Neuroradiology, Anna Meyer Children’s University Hospital, Florence, Italy;

^5^Department of Experimental and Clinical Biomedical Sciences “Mario Serio”, University of Florence, Florence, Italy;

*** Correspondence:**Gilda Belli
[gildabelli@gmail.com](mailto:gildabelli@gmail.com)

| **Metabolic disease** | **Gene (OMIM) Locus** | **Pathogenesis** | **N patients, SEX** | **Clinical features of precocious puberty**  **or pubertal dysfunctions** | **Treatment** | **Possible pathogenic mechanisms** | **Ref.** |
| --- | --- | --- | --- | --- | --- | --- | --- |
| **Mucopolysaccharidosis type III A**  (MPS IIA; SanFilippo A) | **#** 252900  17q25.3  (SGSH; [605270](http://omim.org/entry/605270)) | Lysosomal storage disorder, N-sulfoglucosaminesulfohydrolase deficiency; impaired degradation of heparansulfate | 3 M | Age: 5.9, 6.6 and 9.6 ys; advanced pubertal stage, pubertal levels of basal and GnRH-stimulated LH and FSH, high testosterone levels, no increase in height velocity or bone maturation | GnRH agonist in 2/3, effective | Not investigated/  Discussed | [15] |
|  |  |  | 2 M | Age: 7.6 and 7.7 years, T 6 ml, G2,PH2; no acceleration of growth velocity or bone maturation, high testosterone levels,  positive GnRH stimulation test | GnRH agonist, effective | Triggering role of glycosaminoglycans on glial-neuronal interactions regulating GnRH release (perturbation of critical heparasulfate- cytokine receptor interactions) or  distortion of hypothalamus pathways regulating GnRH release. | [16] |
| **Mucopolysaccharidosis type IH** (MPS 1H;Hurler syndrome) | **#** 607014  4p16.3  (IDUA; [252800](http://omim.org/entry/252800)) | Lysosomal storage disorder,  α-L-iduronidase activity deficiency,  glycosaminoglycans (GAG) heparan and dermatansulfates | 4 | Age: 7.9 e 7.6 years. 1) positive GnRH stimulation test; 2) LH- ICMA 2.0 IU/l, estradiol 69.75 pmol/l); 2 patients had laboratory results consistent with PP, but no clinical signs of puberty | Not reported | Effects of the HSCT or primarly associated (innate) to MPS IH | [17] |
| **GM2-gangliosidosis**  (Tay-Sachs Disease) | **#** 272800  15q23  (HEXA; [606869](http://omim.org/entry/606869)) | Deficiency of alpha subunit of hexosaminidase A, accumulation of monosialic gangliosides 2 (GM2 gangliosides) | 1 F | 4-year-old, enlargement of the mammary glands, then development of pubic hair, vaginal smear, 2 ys and 3 mo later menarche; advanced bone age; normal basal level of FSH e LH, positive LH-RH test | Not treated | accumulation of monosialic gangliosides 2 disrupts LHRH-containing neurons, stimulating gonadotropin release from the pituitary glandor  selective interruption of the inhibitory influences on the pituitary-gonadal axis | [19] |
| **Phenylketonuria** | **#** 261600  12q23.2  (PAH; [612349](http://omim.org/entry/612349)) | Disorder of aminoacids metabolism, deficiency in [phenylalanine hydroxylase](https://ghr.nlm.nih.gov/art/large/ph.jpeg?ow) | 1F | 7.5 years telarche, advanced bone age (9 years). Normal basal values of FSH and LH, positive LHRH stimulation test. Pelvic ultrasonography was normal. Menstruation at the age of 9, despite ciproterone acetate | Cyproterone acetate, poor compliance | Toxic metabolic effect of high serum phenylalanine levels prompts premature activation of the hypothalamic- pituitary axis | [20] |
|  |  |  | 1F | 2.2 ys; breast enlargement, then development of pubic hair, brown vaginal discharge. At 3.2 ys B4, P2, A1, bone age of 4.62 years; positive LHRH test. Pelvic ultrasound: bulky uterus (length 4.75 cm); fundo-cervical-ratio 1.17, endometrial thickness 5.7 mm. Ovaries of normal volume but with large follicle. Values of phenylalanine within recommended range | GnRH agonist. effective | Rare coincidental association (independent by phenylalanine concentrations) | [21] |

**Table 1** Cases of CPP or pubertal disorders in inherited metabolic diseases affecting the brain white matter. Legend: HSCT = Hematopoietic Stem Cell Transplantation; GnRH = gonadotropin-releasing hormone (GnRH).
